# Strengthening Signal Detection in Pharmacovigilance by Using International Nonproprietary Name (INN) Stems

**DOI:** 10.1007/s40264-025-01620-y

**Published:** 2025-10-25

**Authors:** Raffaella Balocco, Jeffrey K. Aronson, Sarel F. Malan, Albert Figueras

**Affiliations:** 1 INN Programme and Classification of Medical Products, World Health Organization (WHO), 20 Avenue Appia, 1211 Geneva, Switzerland; 2 Nuffield Department of Primary Care Health Sciences, Centre for Evidence Based Medicine, University of Oxford, Oxford, United Kingdom; 3 School of Pharmacy, University of the Western Cape, Bellville, South Africa

## Abstract

‘Stems’, which mark pharmacological relationships between substances, form the backbone of the International Nonproprietary Name (INN) system, developed by the WHO in the 1950s. In this paper, we propose using the INN stems to enhance pharmacovigilance signal detection. After analysis of historical cases and current pharmacovigilance practices, we discuss how stem-based classification could facilitate understanding of the adverse-effects profile of each stem, to be used as a benchmark for early identification of adverse drug reactions that deviate from expected class effects, in other words signals associated with newly marketed medicines or different uses of well-known medicines. We propose a potential framework for integrating stem-based analysis into existing pharmacovigilance databases, supplemented by artificial intelligence approaches, such as machine learning. While acknowledging limitations, such as stem variability and reporting bias, we suggest that this approach offers potential advantages for regulatory authorities and healthcare professionals in post-marketing surveillance. Implementation of stem-based post-marketing surveillance could enhance signal-detection efficiency and contribute to improved patient safety through earlier identification of unexpected adverse effects and adverse reactions.

**Supplementary Information:**

The online version contains supplementary material available at 10.1007/s40264-025-01620-y.

## Key Points

**Table:** 

The INN stem is part of the name of a drug, usually as a suffix or an infix.
Stems mark the pharmacological relationship between different substances.
Drugs sharing an INN stem also share many adverse drug reactions which can be used as a benchmark and facilitate the early identification of unexpected reactions.
Analysis based on INN stems could be integrated in the existing pharmacovigilance databases and artificial intelligence approaches for signal detection.

## Introduction

Spontaneous reporting systems remain a cornerstone of post-marketing surveillance of medicinal products (pharmacovigilance) despite inherent limitations, such as under-reporting and the limited amount of clinical data provided in reports. One of the main objectives of pharmacovigilance is early detection of signals, defined as “information that arises from one or multiple sources (including observations and experiments), which suggests a new potentially causal association, or a new aspect of a known association, between an intervention and an event or set of related events, either adverse or beneficial, which would command regulatory, societal or clinical attention, and is judged to be of sufficient likelihood to justify verificatory and, when necessary, remedial actions” [1, 2]. A signal can be either an adverse drug effect or reaction; it may be previously unknown for a specific medicine, a consistent change in the intensity of an adverse drug reaction, or an unusual increase in the frequency of already known and expected adverse effects due to changes in the manufacturing process, preservation or administration conditions, or genetic characteristics of the patients who receive a specific drug [3].

Spontaneous reporting of suspected adverse drug reactions (ADRs) is an extended pharmacovigilance method, used in more than 170 countries who report to the World Health Organization (WHO)’s Programme for International Drug Monitoring (WHO-PIDM) coordinated by the Uppsala Monitoring Centre (UMC) in Sweden [4]. The systems for detecting signals have evolved considerably since manual analyses of received reports were developed in the early 1970s. Electronic databases and advances in managing large amounts of information have enabled improved signal detection through the development of quantitative signal detection methods, particularly disproportionality analyses, which help identify drug-event combinations that are reported more often than expected [5].

Many ADRs occur as a consequence of the structure and mechanism of action of a drug [6, 7]. Such ADRs are also likely to occur in users of another drug with the same mechanism of action or containing similar substructures or functional groups [8]. For example, artery dissections and aneurysms are associated with the use of all vascular endothelial growth factor (VEGF)-inhibitors [9], because VEGF inhibition impairs vascular wall integrity. Tumour necrosis factor (TNF)-α inhibitors can cause or reactivate tuberculosis; this adverse reaction, first detected during use of infliximab, is considered a class effect of TNF-α inhibitors [10]. Quantitative structure-activity relationship (QSAR) modelling has also proven effective in predicting possible adverse effects; substructures like sulfonylarylamine or beta-lactam with adjacent sulfur moieties predicted Stevens-Johnson syndrome.[7] Pharmacological plausibility (i.e., whether an adverse event can be explained through the drug’s known mechanism of action, pharmacokinetic properties, and molecular targets), and biological plausibility (i.e., scientific coherence of the drug-reaction relationship with existing biomedical knowledge, including pathophysiological processes and biological systems) are crucial in pharmacovigilance. Therefore, to understand the adverse-effects profile of a medicine, it is useful to know its mechanism of action and, to some extent, its chemical or biological structure.

Here we propose using INN stems as benchmarks for early signal detection. After describing what stems are and giving examples of substances with the same stem that behave differently, we explore how stems can be integrated into large pharmacovigilance databases, with the aim of accelerating signal detection and preventing adverse reactions.

## The Role of Stems in the International Nonproprietary Name (INN) System

In the 1950s, the WHO developed the International Nonproprietary Name (INN) system to name pharmaceutical substances, guided by two main principles [11, 12]. First, INNs should be distinctive in sound and spelling. They should not be inconveniently long or liable to be confused with names in common use. Secondly, the INN for a substance belonging to a group of pharmacologically related substances should, where appropriate, show that relationship.

In the early years of the INN Programme, it was acceptable to modify chemical names in creating INNs for new drugs. With the discovery of new therapeutic agents whose modes of action could differ, even though they might share similar chemical structures, the chemical approach was eventually superseded by other approaches, structural information being regarded as less informative or useful for prescribers and dispensers. Gradually, with the discovery of more drug targets and better understanding of drug-target interactions, the incorporation of the modes of action in the names of newer drugs became more prevalent. Thereafter, the INN suffixes became indicators of the modes of action or pharmacological classes of the substances. These suffixes became known as ‘stems’ [13]. Typically, an INN consists of a random (‘fantasy’) prefix and a common suffix (‘stem’); sometimes, substems are established to differentiate between different related groups of substances; the INN therefore has the following structure: random prefix + infix + suffix, where infix + suffix = substem.
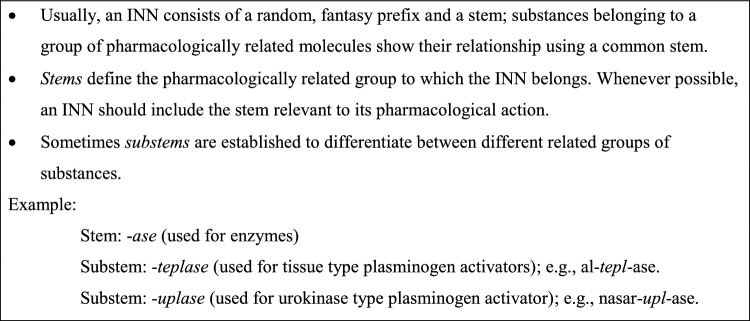


Using stems and substems creates a taxonomic conceptual system for INN and allows users to exploit this systematicity to improve retention, pronunciation, and recognition of the names. For example, ‘montelukast’ contains the stem *-ast* in the substem *–lukast*. Stems and substems have specific definitions, allowing new names to be coined. For montelukast, the definitions are:

-*ast* (anti-allergic or anti-inflammatory, not an antihistamine);

*-lukast* (leukotriene receptor antagonist).

Users can recognise montelukast as a leukotriene receptor antagonist, a medication used to treat asthma; they can also identify other names ending in *–lukast* with similar pharmacological actions [14]. There are currently 14 of these, including gemilukast, pranlukast and zafirlukast.

The relationship between mechanism of action and adverse drug reactions is well established in pharmacology, because drugs that share the same therapeutic target, substructure or biochemical pathway often exhibit similar adverse effects profiles, commonly referred to as ‘class effects’. Therefore, the stem/substem classification system could be used to identify all substances with similar effects on the same target, and also to study shared adverse effects and individual differences.

This differential aspect can be of interest in pharmacovigilance, because knowing the common profile of ADRs associated with certain stems allows distinct, unexpected or unusual reactions to be identified easily and quickly.

For regulatory authorities and healthcare professionals, this classification system provides a framework for anticipating potential ADRs based on known class effects, while remaining alert to variations that might signal unique concerns. Thus, the stem system, especially modern stems, which are for the most part based on the mechanism of action, serves not only as a naming convention but as a foundational element for systematic surveillance of ADRs, offering a structured approach to signal detection.

## Substances, INN Stems, and Adverse Drug Reactions

It is not rare for individual drugs within a class to show a unique adverse reaction pattern, slightly different from that of the other stem members, owing to specific molecular characteristics, minor differences in metabolic pathways, or known or unexpected interactions with secondary targets that are only discovered during large-scale clinical use of the medicine. These cases are of great interest in post-marketing monitoring, because rapid identification and verification of such signals help refine cautions and contraindications; additionally, in certain cases, these differential ADRs require regulatory actions, such as withdrawal of authorisation because of unacceptable toxicity. Table 1 includes some examples.

**Table 1:** Examples of medicines that required regulatory actions because of a differentiated risk compared with other substances sharing the same stem

Stem	Medicine	Adverse drug reaction	Notes
-statin	Cerivastatin	Fatal rhabdomyolysis	The members of the class all have the same mechanism of action and the same ADR, but it is of a greater intensity in a single member of the class compared with the others
-zu-mab	Efalizumab	Progressive multifocal leukoencephalopathy	The same structure and manner of production, but different mechanisms of action and different ADRs from other -*zumabs*
-glitazone	Troglitazone	Liver failure (sometimes fatal)	The same mechanism of action, but one or more members of the class have different ADRs from others in the class
	Rosiglitazone	Cardiovascular risks	
-coxib	Rofecoxib	Cardiovascular events, myocardial infarction	The same mechanism of action, but a spectrum of different intensities of the same ADRs across the class; also, a range of other ADRs to different members of the class
	Valdecoxib	Cardiovascular events, severe skin reactions	
	Lumiracoxib	Liver toxicity	
-oxacin	Trovafloxacin	Fatal liver toxicity	The same mechanism of action, but one or more members of the class have different ADRs from others in the class
	Grepafloxacin	Serious cardiac arrhythmia	
	Ciprofloxacin	QT interval prolongation and torsade de pointes	

Cerivastatin represented a unique case within the statin class, illustrating how molecular differences can create distinct safety profiles. While all substances sharing the *-statin* stem can cause myopathy and rhabdomyolysis, severe muscle toxicity was more frequent with cerivastatin, 3.16 cases of fatal rhabdomyolysis per million prescriptions, unacceptably higher than the 0.19 cases per million seen with other statins. After 52 deaths worldwide were attributed to cerivastatin-induced rhabdomyolysis, it was withdrawn in 2001 [15]

Troglitazone is a compelling example of how subtle molecular differences between substances sharing the same stem can lead to concerns about harms. It belongs to the stem *-glitazone* (peroxisome proliferator activating receptor-γ (PPAR-γ) agonists, thiazolidinedione derivatives). It was approved in 1997, and it soon became apparent that it had a unique hepatotoxicity profile not seen with other -*glitazones*, like rosiglitazone and pioglitazone. Troglitazone was withdrawn in 2000 after reports of 90 cases of liver failure, with several deaths or patients requiring liver transplantation, while other members of the stem remained on the market with acceptable patient safety profiles [16].

Efalizumab exemplifies how biologics sharing the same stem and substem (*-zumab* for humanized monoclonal antibodies) can exhibit unique concerns. While all immunosuppressive biologics carry inherent risks of opportunistic infections, efalizumab posed unprecedented concern, because it was associated with progressive multifocal leukoencephalopathy (PML). Between 2003 and 2009, four confirmed cases of PML emerged among approximately 46,000 treated patients. This risk was noteworthy because PML had not previously been associated with psoriasis treatments. The drug was voluntarily withdrawn from global markets in 2009 [17].

The stem *-coxib* (selective cyclo-oxygenase inhibitors) is an interesting example that shows how drugs within a stem can have varying degrees of risks for class-related adverse reactions. These drugs were developed and marketed as having a lower gastrointestinal risk than non-selective inhibitors, which is true. These new drugs were therefore promoted among users of non-steroidal anti-inflammatory drugs (NSAIDs), including elderly people. This rapidly increased use of new medicines led to detection of unexpected and serious ADRs that led to the withdrawal of some selective inhibitors and a call to use both selective and non-selective COX inhibitors carefully [18–22].

The stem -*oxacin* (antibacterials, nalidixic acid derivatives) includes fluoroquinolone antibacterials. Although they have the same mechanism of action, one or more members of the class have different ADRs to others in the class; trovafloxacin was withdrawn from the market because of fatal liver toxicity [23], and grepafloxacin because of serious cardiac arrhythmia [24].

In cases like these, substances have been withdrawn from the market after causing several deaths. In contrast, in other cases, the confirmation of a signal has led to modification of the recommended dosage regimen, including contraindications or advice to avoid certain concomitant treatments.

In contrast, ciclosporin and tacrolimus have different stems, but both are calcineurin inhibitors with immunosuppressant effects and a similar adverse reaction profile. Sirolimus shares its stem with tacrolimus, but it inhibits the mTOR (mammalian target of rapamycin); their safety profile is quite comparable, dominated by the immunosuppressant effects.

Pharmacovigilance aims to learn from these cases and improve detection systems, to accelerate the spotting of unexpected adverse reactions and to prevent as many deaths as possible. The crucial aspect is that a broad knowledge of the shared safety profile of the stem can help to speed up identification of any adverse effects pattern that significantly differs from the expected one.

## Expanding the Use of INN Stems in Pharmacovigilance

INN stems could facilitate efficient analysis of pharmacovigilance databases, such as the WHO’s VigiBase or the US FDA’s FAERS (FDA Adverse Event Reporting System). Researchers and regulators can identify class-specific ADR patterns and detect emerging signals by querying these databases using INN stems. These types of queries are not automated and usually require retrieval of large amounts of information for individual drugs requiring several database consultations to obtain all the information about medicines that share a stem. Therefore, implementing a stem-based surveillance approach requires, first and foremost, that large pharmacovigilance databases allow information retrieval by grouping suspected medicines included in the reports, not only by the INN and the Anatomical Therapeutic Chemical (ATC) classification code, but also by the stem or substem (e.g., “reports involving a -coxib”, or “reports involving a -meran”). This could be achieved by linking the INN with stems and substems in the database, as well as cross-referencing stems, INN and ATC to facilitate searches.

Machine learning and artificial intelligence will probably play crucial roles in enhancing analysis of pharmacovigilance data, by identifying patterns and trends associated with specific INN stems. These technologies could be particularly valuable in processing vast amounts of data generated through stem-based queries, once the databases are ready to provide results grouped by stem. Furthermore, natural language processing (NLP) algorithms can extract ADR information directly from electronic health records (EHRs) and social media, providing real-world evidence to complement traditional pharmacovigilance methods.

For example, a recent study [25] showed how systematic analysis of healthcare registry data could have identified the cardiovascular risks of rofecoxib much earlier, through sequential monitoring using case-time-control and cohort study designs. The authors found that the risk of myocardial infarction could have been detected 3.5 years before the market withdrawal of rofecoxib, potentially preventing many adverse events. This method could be effectively integrated with a stem-based pharmacovigilance approach. By combining sequential epidemiological analyses with INN stem classification, it could be possible to create a more robust early warning system: stems would provide the initial framework for expected adverse reaction profiles based on pharmacological class, while sequential analyses would provide statistical validation of any detected signals. In future, integrated approaches could use both pharmacological aspects and real-world data, potentially enabling faster identification of safety signals, especially for newly marketed drugs for which early detection is crucial.

From the perspective of regulatory authorities, immediately after authorisation of any new product, pharmacovigilance teams can start a living process to compare the profile of reported ADRs with the expected ones according to the INN stem of the new drug, thus facilitating identification of any significant deviation from the expected pattern. This can improve updates of clinical recommendations addressed to healthcare professionals and the population.

For manufacturers, early identification of potential risks with the aid of large database analyses can be highly valuable to avoid over-advertising the use of a drug for a population at risk or in dosages that could increase the risks of certain outcomes.

The online resource includes a case study using the US FAERS database to analyse the example of optic neuropathy and the *-tide* stem (e.g., semaglutide, exenatide, octreotide) (see Online Supplemental Material, Case study).

## Limitations and Challenges

Despite the theoretical advantages of this approach, it is important to highlight certain limitations. A significant challenge lies in the variability of certain stems, especially those introduced at the beginning of the INN Programme. This situation can impede analysis of the ‘stem’ ADR profile as a benchmark for new drugs of the stem, for example, in the case of stems based on the chemical structure but mixing different mechanisms of action. For example, beta-blockers mostly have the same stem, *-olol*, but not all of them (labetalol and carvedilol being exceptions). Stanozolol, on the other hand, is not a beta-blocker. Even among the beta-blockers with –olol there are wide differences in actions, for example, adrenoceptor selectivity, partial agonism, lipophilicity, and therefore different adverse effects in the brain [26]. This illustrates the limitations of certain stems assigned according to the chemical structure.

Moreover, the first marketed medicine with a new stem cannot benefit from this approach, as no comparative data exist within the stem class. This is also valid for biologics, as certain mechanisms of action are not well understood when the INN is given (typically when only phase 1 clinical trials have been conducted), and the classification can evolve as new substances are discovered. The *-mab* stem is a good example of the evolution of naming.

A special case is presented by biosimilars, which, while having the same stem or substem, may nevertheless, because of differences in glycosylation, have different pharmacokinetics and different adverse effects. For example, a recent study comparing biosimilars to originators found that adalimumab biosimilars showed higher reporting rates for certain adverse events like injection site pain and arthralgia compared to the originator product. The authors attributed these differences to different factors, such as a small degree of controlled variability, so that, although biosimilars have the same amino acid sequence, they may have different glycosylation patterns or other structural variations, because of production in different cell lines [27]. The INN Expert Group has proposed the use of a Biological Qualifier (BQ) to address this issue in different opportunities – though it has not yet been applied and remains pending [28].

The implementation of stem-based pharmacovigilance would require adapting database searches and filter tools to link them to their stems. Integrating stem-based searching capabilities into existing systems requires resources and coordination among stakeholders. Other classifications, such as the ATC, although useful for certain research and administrative purposes, has limitations concerning the grouping of drugs, because many ATC subgroups include an ‘Others’ section with a mixture of substances with different stems and mechanisms of action.

Pharmacovigilance schemes based on reporting ADRs face important and well-known limitations: under-reporting, reporting bias, and low-quality reporting, which are due to many factors. For example, the novelty of a medicine or the calling effect of news and social media can explain reporting biases, leading to clusters of reported suspected events, and factors such as lack of knowledge, fear of reporting, and lack of time can explain under-reporting [29]. Finally, not all reports are detailed enough to attribute causality appropriately; in some settings, more than one-quarter of the reports are of low-quality and cannot be used for disproportionality calculations [30]. Furthermore, grouping has a potential impact on disproportionality calculations [31], and using INN could help overcome certain effects by guiding which medicines can be selected for comparisons.

Successful implementation of stem-based pharmacovigilance requires these limitations to be addressed, while maintaining system efficiency and reliability. This includes developing appropriate statistical thresholds for stem-specific signals, handling combination products and complex biologics effectively, and integrating newer therapeutic modalities into the system. Continuous evaluation and refinement of the approach will be necessary as new challenges emerge, and pharmacovigilance practices evolve.

## Conclusion

A systematic stem-based approach to pharmacovigilance could enhance current adverse reaction monitoring analyses of large databases. By harnessing the inherent relationship between INN stems and mechanisms of action and mapping the adverse reaction profile of each stem, this approach offers a benchmark for detecting unexpected signals of new medicines or changes in the frequency of already known adverse reactions to drugs used in different circumstances or populations. Historical examples demonstrate how drugs within a stem class can occasionally exhibit unique patient safety concerns that deviate from expected class effects. As pharmaceutical development continues to advance, and increasingly complex therapeutic modalities enter the market, this stem-based approach, integrated with database searching tools, could provide valuable insights for pharmaceutical companies, regulatory authorities, and healthcare professionals, ultimately contributing to more effective post-marketing surveillance and patient safety.

## Supplementary Information

Below is the link to the electronic supplementary material.

Supplementary file1 (PDF 248 KB)
