# Supplementary material for: Strengthening Signal Detection in Pharmacovigilance by Using International Nonproprietary Name (INN) Stems
Source: Drug Saf. 2025 Oct 25;49(4):381–7. doi: 10.1007/s40264-025-01620-y (PMC13002644; doi:10.1007/s40264-025-01620-y)
Supplement: Supplementary file 1 — Supplementary file1 (PDF 248 KB) [file 40264_2025_1620_MOESM1_ESM.pdf]

Electronic Supplementary Material

Title: Strengthening signal detection in pharmacovigilance by using International Nonproprietary Name (INN) stems

Journal: Drug Safety

Authors:

Raffaella Balocco, INN Programme and Classification of Medical Products, World Health Organization (WHO), Geneva, Switzerland. ORCID: 0000-0002-1358-1743

Jeffrey K. Aronson, Centre for Evidence Based Medicine, Department of Primary Care Health Sciences, University of Oxford, United Kingdom. WHO INN Expert. ORCID: 0000-0003-1139-655X

Sarel F. Malan, School of Pharmacy, University of the Western Cape, Bellville, South Africa. WHO INN Expert. ORCID: 0000-0002-1640-6457

Albert Figueras, Consultant to the INN Programme and Classification of Medical Products, World Health Organization (WHO), Geneva, Switzerland. ORCID: 0000-0002-2740-2013

Corresponding Author: Albert Figueras, email: figuerasa@who.int. INN Programme and Classification of Medical Products, World Health Organization, 20 Avenue Appia, 1211Geneva, Switzerland.

Case study: Substem-based signal detection in FAERS - Optic neuropathy and *-tide* stem analysis

1. Background and methods

This analysis was prompted by recent European Medicines Agency (EMA) safety communications regarding non-arteritic anterior ischemic optic neuropathy (NAION) associated with the use of semaglutide.

Objective. To test the practical application of INN stem- and substem-based pharmacovigilance for detecting individual case safety signals (ICSRs) while controlling for confounding by indication.

Database and extraction. We conducted a disproportionality analysis using the FDA Adverse Event Reporting System (FAERS) database with data extraction performed on 4 August 2025.

Adverse reactions definition: We selected five related optic nerve disorders: "optic ischaemic neuropathy," "optic nerve disorder," "optic nerve infarction," "optic neuritis," and "optic neuropathy."

Study population: From 24,447 ICSRs reporting these optic nerve events, we identified 413 cases attributed to drugs with "-tide" substems. The reference population comprised 486,774 ICSRs for all *-tide* drugs, yielding an overall background rate of 0.848 per 1000 ICSRs (413/486,774).

Drug classification: The analysis focused on seven major *-tide* substems: *-clotide* (guanylate cyclase agonists); *-enatide* (GLP-1 receptor agonists); *-glutide* (GLP-1 receptor agonists); *-paratide* (parathyroid hormone analogues); *-patide* (GIP receptor agonists); *-reotide* (somatostatin analogues), and *-ritide* (natriuretic peptides).

The *-tide* stem includes other substems (*-actide* [synthetic polypeptides with a corticotropin-like action]; *-dutide* [dual agonists at GLP-1 receptors and glucagon receptors]; *-motide* [agents for immunization]; *-netide* [agents used in neurology]; and *-pultide* [pulmonary surfactants]), but these substems have not been included in the analysis because the database did not include any report describing ocular toxicity in patients taking any of these drugs.

Statistical methods: We calculated Rate Ratios (RR) as drug-specific rates divided by the overall background rate, with RR ≥3.0 considered a signal of disproportionate reporting (SDR). Sensitivity analyses included substem-level aggregation and individual drug assessment. [1,2]

2. *Main findings*

Primary signal detection: Tables 1 and 2 describe the main findings. Three drugs exceeded the statistical threshold: semaglutide (6.414 per 1000 ICSRs, RR = 7.6), octreotide (3.416 per 1000 ICSRs, RR = 4.0), and lanreotide (3.831 per 1000 ICSRs, RR = 4.5). At the substem level, *-reotide* showed the highest rate (2.952 per 1000 ICSRs), followed by *-glutide* (1.900 per 1000 ICSRs).

Table 1: Substem-based optic neuropathy (ON) Reporting Rates in FAERS analysis.

Substem	Representative drugs	Total ICSRs	ON Cases	Rate per 1000 ICSRs	RR**	Clinical validation
**-reotide**	octreotide, lanreotide	4,742	14	2.952	3.5	**Confounding by indication** - 20-43% baseline visual complications in acromegaly
**-glutide**	semaglutide, liraglutide, dulaglutide	172,270	327	1.900	2.2	**Mixed signals** - Semaglutide confirmed (RR = 7.6) others near background
**-ritide**	vosoritide, nesiritide	1,416	1	0.706	0.8	No established association
**-patide**	tirzepatide	82,920	42	0.507	0.6	No established association
**-clotide**	linaclotide	14,407	2	0.139	0.2	No established association
**-paratide**	teriparatide, abaloparatide	131,623	18	0.137	0.2	No established association
**-enatide**	exenatide, lixisenatide	79,396	7	0.088	0.1	No established association

*ICSRs = individual case safety reports. ON = "optic neuritis" (see Methods); RR = rate ratio.
**RR calculated as drug-specific rate divided by background rate (0.848 per 1000 ICSRs); RR ≥3.0 indicates signal of disproportionate reporting (SDR).

Table 2: Detailed analysis of *-glutide* substem drugs - Optic Neuropathy reporting in FAERS

Drug	Total ICSRs	Optic Neuropathy Cases	Rate per 1000 ICSRs	RR*	RR vs Substem	Clinical Status	Regulatory Actions
**semaglutide**	45,056	289	6.414	**7.6**	**3.4**	**Confirmed SDR**	EMA/FDA safety communications (2024); enhanced monitoring required
**liraglutide**	38,339	24	0.626	0.7	0.3	No established association	No regulatory actions
**dulaglutide**	72,254	13	0.180	0.2	0.1	No established association	No regulatory actions
**teduglutide**	7,983	1	0.125	0.1	0.1	No established association	No regulatory actions
**albiglutide**	8,637	0	0.000	0.0	0.0	No established association	Withdrawn worldwide in 2018 (commercial reasons)
**elsiglutide**	1	0	0.000	0.0	0.0	Insufficient data	Investigational

*Rate Ratio (RR) vs background rate (0.848 per 1000 ICSRs)
**RR vs substem background rate (1.900 per 1000 ICSRs)

Case-by-case assessment through scientific literature validation: Following READUS-PV recommendations, we performed a scientific literature review and regulatory database consultation in a surrogate case-by-case analysis. This revealed fundamentally different aetiologies for apparent signals. For semaglutide, the signal represented a validated PV finding with recently established regulatory recognition, published clinical evidence,[3] and plausible biological mechanisms involving GLP-1 receptors in retinal ganglion cells. The drug-specific pattern within the GLP-1 class (other *-glutide* drugs showed much lower rates) supported genuine causality.

Confounding by indication identified: In contrast, the somatostatin analogue signals (*-reotide* substem) exemplified classic confounding by indication. Comprehensive investigation revealed:
(1) no published cases of somatostatin analogue-induced optic neuritis despite decades of clinical use;

(2) absence of regulatory safety signals across major pharmacovigilance databases;
(3) 20-43% baseline prevalence of visual field defects in patients with acromegaly before the start of treatment, and
(4) documented cases in which visual symptoms preceded drug treatment, with somatostatin analogues prescribed specifically for "rescue of vision."

Clinical context and disease-related confounding

The analysis highlighted how underlying disease pathology can masquerade as drug-induced adverse events. Patients with acromegaly (a primary indication for octreotide/lanreotide) often present with pituitary macroadenomas causing direct optic chiasm compression, resulting in visual field defects in 20-43% of patients at diagnosis. Similarly, other indications for somatostatin analogue—neuroendocrine tumours, Cushing's disease, and carcinoid syndrome—carry inherent risks of visual complications through mechanisms including paraneoplastic optic neuropathy, nutritional deficiencies, and compressive effects.

3. Methodological implications

This case study suggests critical principles for stem/substem-based PV:

(1) statistical signals require comprehensive clinical validation;
(2) baseline disease characteristics must be considered when interpreting signals of adverse drug reactions;
(3) temporal analysis should include pre-treatment adverse event status, and
(4) the treatment context provides essential diagnostic clues for signal interpretation.

This highlights the importance of a careful causality analysis and facilitate grouping reports according to the temporal sequence or the existence of alternative causes.

The contrasting findings between semaglutide (confirmed signal) and somatostatin analogues (confounding by indication) using identical statistical thresholds emphasize that disproportionality analysis represents only the initial step in signal evaluation. Effective substem-based pharmacovigilance requires integration of statistical detection with systematic literature review, regulatory database analysis, temporal relationship assessment, and biological plausibility evaluation.

4. Pharmacovigilance implications

This heterogeneity within the *-glutide* substem suggests that substem-based analysis can identify both class-wide patterns and individual drug deviations. The stark contrast between semaglutide and other GLP-1 agonists strengthens the causal inference for semaglutide specifically, as it rules out a general class effect and points to drug-specific characteristics as the likely mechanism.

The findings support targeted safety monitoring for semaglutide while maintaining standard surveillance for other *-glutide* drugs, illustrating how substem-based approaches can guide risk-stratified pharmacovigilance strategies.

It should be noted that semaglutide (ATC code A10BJ06) is classified under the ATC4 subgroup A10BJ (Glucagon-like peptide-1 (GLP-1) analogues), which also includes exenatide, liraglutide, lixisenatide, albiglutide, and dulaglutide. However, tirzepatide (A10BX16) is classified as A10BX (Other blood glucose lowering drugs, excl. insulins). Concerning other substems, octreotide, for example, is under H01CB (Somatostatin and analogues). Therefore, searches based only on ATC codes risk including slightly different substances or excluding related substances classified under other pharmacological or therapeutic groups in other organ/system levels.

References

1. Fusaroli M, Salvo F, Begaud B, *et al*. The Reporting of a Disproportionality Analysis for Drug Safety Signal Detection Using Individual Case Safety Reports in PharmacoVigilance (READUS-PV): Development and Statement. *Drug Saf*. 2024; 47:575-584.

2. Hauben M, Aronson JK. Defining 'signal' and its subtypes in pharmacovigilance based on a systematic review of previous definitions. *Drug Saf.* 2009;32(2):99-110. doi: 10.2165/00002018-200932020-00003.
3. EMA. PRAC concludes eye condition NAION is a very rare side effect of semaglutide medicines Ozempic, Rybelsus and Wegovy. 6th June 2025. https://www.ema.europa.eu/en/news/prac-concludes-eye-condition-naion-very-rare-side-effect-semaglutide-medicines-ozempic-rybelsus-wegovy (last access: 21 August 2025)
